# Fine mapping of *Brassica napus* blackleg resistance gene *Rlm1* through bulked segregant RNA sequencing

**DOI:** 10.1038/s41598-019-51191-z

**Published:** 2019-10-10

**Authors:** Fuyou Fu, Xunjia Liu, Rui Wang, Chun Zhai, Gary Peng, Fengqun Yu, W. G. Dilantha Fernando

**Affiliations:** 1 Saskatoon Research Centre, Agriculture and Agri-Food Canada, 107 Science Place, Saskatoon, Saskatchewan S7N 0X2 Canada; 2 Department of Plant Science, University of Manitoba, Winnipeg, MB R3T 2N2 Canada; 3 Chongqing Engineering Research Center for Rapeseed, College of Agronomy and Biotechnology, Southwest University, Chongqing, 400716 China

**Keywords:** Agricultural genetics, Agricultural genetics, Biotic, Biotic

## Abstract

The fungal pathogen *Leptosphaeria maculans* causes blackleg disease on canola and rapeseed (*Brassica napus*) in many parts of the world. A *B*. *napus* cultivar, ‘Quinta’, has been widely used for the classification of *L*. *maculans* into pathogenicity groups. In this study, we confirmed the presence of *Rlm1* in a DH line (DH24288) derived from *B*. *napus* cultivar ‘Quinta’. *Rlm1* was located on chromosome A07, between 13.07 to 22.11 Mb, using a BC_1_ population made from crosses of F_1_ plants of DH16516 (a susceptible line) x DH24288 with bulked segregant RNA Sequencing (BSR-Seq). *Rlm1* was further fine mapped in a 100 kb region from 19.92 to 20.03 Mb in the BC_1_ population consisting of 1247 plants and a F_2_ population consisting of 3000 plants using SNP markers identified from BSR-Seq through Kompetitive Allele-Specific PCR (KASP). A potential resistance gene, *BnA07G27460D*, was identified in this *Rlm1* region. *BnA07G27460D* encodes a serine/threonine dual specificity protein kinase, catalytic domain and is homologous to *STN7* in predicted genes of *B*. *rapa* and *B*. *oleracea*, and *A*. *thaliana*. Robust SNP markers associated with *Rlm1* were developed, which can assist in introgression of *Rlm1* and confirm the presence of *Rlm1* gene in canola breeding programs.

## Introduction

Blackleg (stem canker), caused by the ascomycete fungus *Leptosphaeria maculans* (Desmaz.) Ces. et De Not. [anamorph: *Phoma lingam* (Tode ex Fr.) Desmaz.], is one of the most important diseases of canola and rapeseed in many parts of the world^1,2^. It has been suggested that *L*. *maculans* can cause severe infection in a field and reduce the seed yield by more than 50%^3^. Improving resistance to *L*. *maculans* is one of the major objectives in canola breeding programs worldwide, especially due to the fact that the disease is difficult to control with fungicides^4^.

Efforts have been made to identify and map blackleg *R* genes in *B*. *napus*. The *R* genes *LEM1*^5^, *LmFr1*^6^, *LmR1*/*CLmR1*^7,8^, *Rlm1*, *Rlm3*, *Rlm4*, *Rlm7* and *Rlm9*^6–9^ have been mapped to the *B*. *napus* linkage group A07, and *Rlm2* to A10^9^. Four additional *R* genes, *LepR1*, *LepR2*, *LepR3* and *LepR4*, originating from an accession of wild *B*. *rapa* subsp. *sylvestris*, have been identified in *B*. *napus* or progenies from *B*. *napus* x *B*. *rapa* subsp. *sylvestris*. *LepR1* and *LepR2* were mapped to *B*. *napus* linkage groups A02 and A10, respectively^10^. *LepR3*, derived from *B*. *rapa* subsp. *sylvestris* and present in the *B*. *napus* cultivar Surpass 400, was mapped to the *B*. *napus* linkage group A10^11^. *LepR3* and *Rlm2* have been cloned with a map‐based cloning strategy, and encode a receptor-like protein (RLP)^12,13^. Blackleg *R* genes previously mapped in *B*. *napus* are generally the dominant type, except *LepR4* (*Brassica* A-genome), which was mapped to the *B*. *napus* linkage group A06^14^. The *R* genes *BLMR1* and *BLMR2* were mapped to the *B*. *napus* linkage group A10 in the *B*. *napus* cultivar Surpass 400^15^. All these blackleg *R* genes were identified through genetic mapping with a marker system, such as RFLPs, Simple Sequence Repeats (SSRs), Amplified Fragment Length Polymorphisms (AFLPs), Cleaved Amplified Polymorphic Sequences (CAPSs), or Sequence Related Amplified Polymorphisms (SRAPs). Developing and genotyping these types of molecular marker can be laborious and time consuming.

Bulk segregant analysis (BSA), which consists of genotyping two bulks of individual plants with extreme phenotypes, was developed to rapidly identify genetic markers linked to genomic regions associated with selected phenotypes^16^. Presently, the pipelines of BSA and high throughput sequencing have been developed to locate and identify candidate genes associated with selected phenotypes, using BSA RNA sequencing (BSR-Seq)^17–19^.

Canola is the most important oilseed crop in Canada. ‘Quinta’, a winter oilseed rape originating from Europe, was found to be highly resistant to lots of Canadian isolates and was widely used as a differential cultivar for the classification of pathogenicity groups^20–24^. The first genetic evidence for a gene-for-gene interaction between *Brassica* and *L*. *maculans* was observed between the resistance (*R*) gene *Rlm1* in ‘Quinta’ and the corresponding avirulence gene *AvrLm1*.^25^. Delourme *et al*. (2004) roughly mapped *Rlm1* in ‘Quinta’ to linkage group A07. The *R* gene was further mapped using SSR and CAPS markers in a large genetic interval^26^.

In western Canada, *AvrLm1* was detected in 46% of the *L*. *maculans* isolates collected from infested canola stubble between 1997 and 2005^27^. Although recent data showed lower presence of *AvrLm1 in L*. *maculans* populations^28,29^ due to use of *Rlm1* in canola cultivars extensively^30^, *Rlm1* could still be valuable for effective control of blackleg disease in Canada as a strategy through rotation of R genes that has been recently implemented.

The objectives of the current study were to: (i) identify genome wide DNA variants to map *Rlm1* through BSR-Seq; (ii) determine the precise location of *Rlm1*; (iii) identify the most probable candidates for *Rlm1*; and (iv) develop SNP markers tightly linked to *Rlm1* in use of marker assisted selection. In this study, *Rlm1* was located on chromosome A07 using a modified popoolation2 pipeline withBSR-Seq. To the best of our knowledge, this is the first report on mapping of a blackleg resistance gene using BSR-Seq in *Brassica* spp..

## Materials and Methods

### Plant materials

Two doubled haploid lines, DH24288 (resistant, R) from the *B*. *napus* cultivar ‘Quinta’ and DH16516 (susceptible, S) from *B*. *napus* cultivar ‘Topas’, were kindly provided by Dr. G Séguin-Swartz at Saskatoon Research and Development Centre, Agriculture and Agri-Food Canada. A DH24288 plant (male) was crossed with a DH16516 plant (female) to produce a F_1_ population. A F_1_ plant was backcrossed with a DH16516 plant to obtain a BC_1_ population. The F_1_ plant was self-pollinated to produce a F_2_ population.

### Plant growth conditions, preparation of *L*. *maculans* isolates and plant inoculations

*L*. *maculans* inoculum and plant inoculation followed the protocols described previously^10^. The parents, F_1_, F_2_, and BC_1_ were inoculated with isolate *L*. *maculans* SC006 (carrying *AvrLm1*). The reaction to inoculation on cotyledon was rated 10 to 14 days post-inoculation (dpi) using the 0 to 9 scale described by Williams^31^. Disease ratings of 0 to 5 and 6 to 9 were considered as resistant and susceptible interactions, respectively. Segregation for resistance and susceptibility in the BC_1_ population was analyzed using the Chi-square (χ^2^) test for goodness-of-fit^32^. *B*. *napus* susceptible cultivar ‘Westar’ was used as a susceptible check in this study.

### RNA isolation and RNA-seq

The BC_1_ population was used for RNA-Seq. At 10 dpi, leaf samples from 30 R plants and 30 S plants were combined to form R and S bulks, respectively; each bulk was treated as one biological replicate. Three replicates, consisting of a total of 90 R or 90 S plants, were used. RNA from each sample replicate was isolated using an RNeasy Plant Mini Kit (Qiagen; Toronto, ON) with on-column deoxyribonuclease (DNase) digestion using a Qiagen RNase-Free DNase kit, following manufacturer’s instructions. The RNA concentration and quality were checked using a NanoDrop 2000c Spectrophotometer (Thermo Scientific; Waltham, MA) and an Agilent Bioanalyzer 2100 (Agilent Technologies; Mississauga, ON), to ensure that the RNA integrity number (RIN) was >8 for each sample. The preparation of cDNA library and RNA-seq were performed at Databio2 LLC (Ames, IA).

### Sequence alignment, SNP calling and filtration

Raw RNA-Seq reads were trimmed to remove low-quality nucleotides with FASTX-Toolkit (Version 0.0.13)^33^. GSNAP (Genomic Short-read Nucleotide Alignment Program, version 2016-11-07)^34^ was performed to map the trimmed reads to the *B*. *napus* reference genome^35^, allowing gap alignment including intron-spanning alignment. SNP calling and filtration for BSR-Seq was performed following the same parameters as previously described by Liu *et al*.^17^. The SNP discovery pipeline (123SNP) was downloaded from http://schnablelab.plantgenomics.iastate.edu/software/123SNP/. Reads of the R and S bulks were more than 90% of the total reads aligned to each SNP site. Each SNP site had ≥3 reads with a quality score of SNP base ≥15, and the reads accounted for 20% of the total reads at that SNP site. Moreover, each SNP site for the BSR-Seq analysis was required to have at least five sequencing reads in both the R and S bulks. In addition, two methods for SNP calling were used as previously described^18^; the single sample alignment (SSA), in which short reads from three biological replicates of R and S plants were aligned to a reference genome. The other was called the pooled sample alignment (PSA), in which short reads from a pool of three R bulks and a pool of three S bulks were aligned to the reference genome as described by Yu *et al*.^18^.

### Identification of SNP markers tightly linked to *Rlm1*

**Figure 1:**
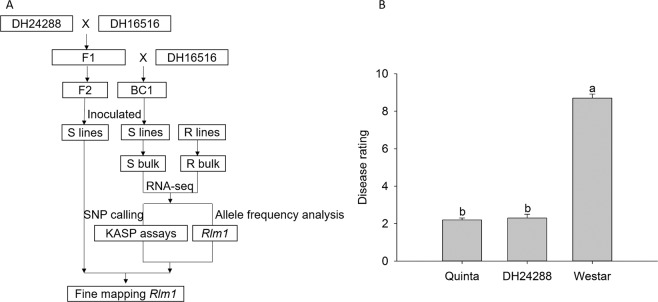
Schematic flowchart and disease ratings. (**A**) Schematic flowchart of the experimental procedure. (**B**) Average disease ratings of *B*. *napus* cultivars ‘Quinta’ and its DH line DH24288. Westar is the susceptible control. The *L*. *maculans* isolate SC006 carries *AvrLm1*. The disease reaction on cotyledons was rated at 10 dpi using the 0 to 9 scale^31^. Mini bars indicate the standard deviation from three biological replicates of three independent repetitions (n = 9). Letters indicate significant difference based on the Least Significant Difference (LSD) (P < 0.05).

In order to verify SNP markers and the target region, an improved PoPoolation2^36^ was employed to analyze the linked SNP markers. (Fig. 1A). Firstly, the same files of R and S bulks were obtained as previously described using GSNAP^34^. Then, Picard tools (Version 1.96, http://broadinstitute.github.io/picard/) was performed to remove duplicate and ambiguously mapped reads. Finally, the allele frequencies of R and S bulks were compared using PoPoolation2^36^. All analyses used the SSA and PSA methods described previously^18^ with reference *B*. *napus* Darmor-bzh genome (v4.1,http://www.genoscope.cns.fr/brassicanapus/data/)^35^.

### Identification of *Rlm1* candidate genes

In order to identify the candidates for *Rlm1* in the target region, all genes in this region were analyzed using the following steps:

1. We annotated all proteins to the target region using InterProScan^37^.
2. The variation of R genes in the target region was identified using samtools^38^ and SNPeff 4.1^39^. The variant frequency of *Rlm1* was approximately 0.5 in the R bulks and 1 in S bulks, since the resistance genotype would be *Rr* and susceptible genotype be *rr* in the BC_1_ population.
3. Resistance gene prediction

The plant resistance genes were identified using RGAugury pipeline^40^ with reference B. napus Darmor-bzh genome (v4.1,http://www.genoscope.cns.fr/brassicanapus/data/)^35,41^.

### Gene expression analysis using RNA-Seq

All trimmed RNA-seq reads were aligned^18^ with reference *B*. *napus* Darmor-bzh genome (v4.1,http://www.genoscope.cns.fr/brassicanapus/data/)^35^ using Tophat 2^42,43^. Tophat2 alignment parameters were set to allow a maximum of two mismatches and exclude reads mapping to more than one position on the reference. Transcriptome assembly and differential expression analysis for RNA-Seq were conducted using cufflink, with FDR < 0.05 and minimum fold change ≥2.

### Genotyping SNP and InDel markers

Selected SNPs identified in the target region were confirmed using the Kompetitive Allele Specific PCR (KASP) method (http://www.lgcgroup.com), following manufacturer’s instructions. The primer sequences of KASP assay were presented in Table S1. The PCR reaction was performed using StepOne Plus Real Time PCR system (Applied Biosystem, Mississauga, ON).

### Phylogenetic tree construction and sequence alignment

The protein sequences of *STN7* were searched using Blastp with default parameters in the nr database of NCBI. Alignment was performed using MAFFAT^44^ with distance correction off, gaps excluded. Then the alignment sequence computed the residue-wise confidence scores and extracted well-aligned residues using GUIDANCE2^45^. The neighbor-joining (NJ) clustering method^46^ was performed to build a phylogeny tree with bootstrap 1000. Visualization was performed using Interactive tree of life (iTOL) v4 (https://itol.embl.de/)^47^.

## Results

### Identification of *Rlm1* in the *B*. *napus* DH line DH24288

DH24288 was derived from a single microspore of the cultivar ‘Quinta’ (*B*. *napus*). Its resistance to the *L*. *maculans* isolate SC006 (*AvrLm1*) was compared with its donor (Quinta) and the susceptible cultivar ‘Westar’ (Fig. 1B) based on cotyledon inoculation. As expected, ‘Westar’ was highly susceptible whileDH24288 and ‘Quinta’ showed a high level of resistance, implying that DH24288 carries *Rlm1*.

### RNA-Seq and sequence alignment

**Figure 2:**
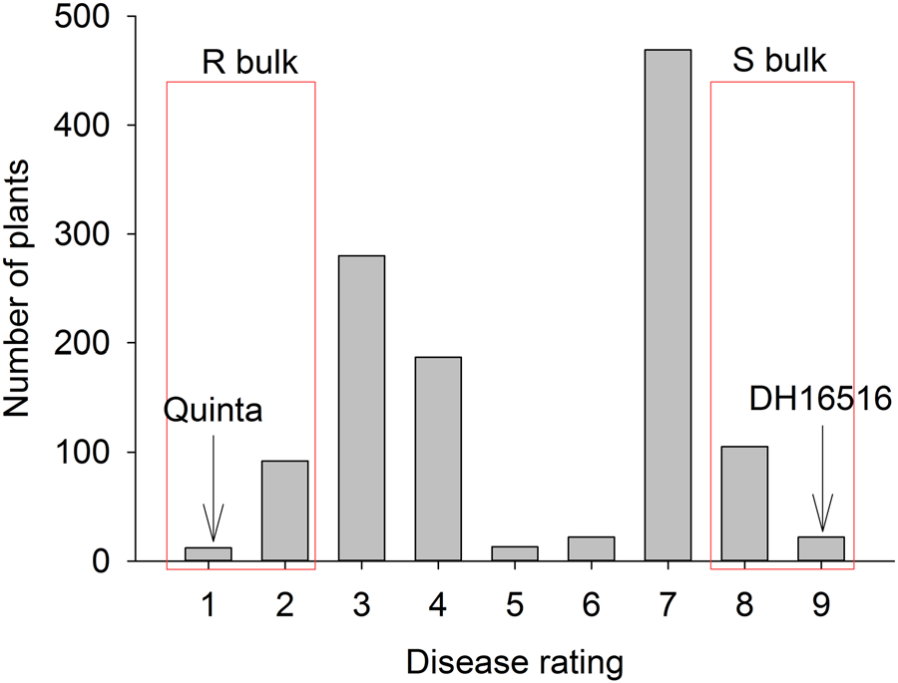
Frequency distributions of resistance phenotypes in the BC_1_ population derived from DH16516 x DH24288/’Quinta’. Parents and BC_1_ plants were inoculated with *L*. *maculans* isolate SC006. The symptoms on cotyledons was rated at 10–14 dpi using the 0 to 9 scale^31^. Disease ratings of 0 to 4 and 5 to 9 were considered as resistant and susceptible interactions, respectively. The lines in the red boxes were selected as R and S bulks for RNA-seq analysis.

In order to identify the resistance gene in the DH line, we tested the BC_1_ segregating population and their parental lines with the isolate SC006. DH16516 was highly susceptible (Fig. 2). The BC_1_ population consisted of 571 R and 596 S plants, which would fit a 1:1 ratio (χ^2^ = 0.54, P = 0.46). Furthermore, the F_2_ population showed 2,216 R and 696 S plants, which would fit a 3:1 ratio (X^2^ = 1.88, P = 2.37), confirming that *Rlm1* is a single dominant gene in DH24288. A total of 180 plants with a clear R (DR ≤ 2) and S (DR ≥ 8) designation, consisting of 90 R and 90 S (Fig. 2) were selected from the population for mapping of *Rlm1* using BSR-Seq.

More than 90 million 101 paired-end reads were obtained, including 47 million from R bulks and 45 million from S bulks. After quality control and clipping of adapters, more than 99% of the reads were retained for both bulks, with the average length reduced to about 98 bp (Table S2).

**Figure 3:**
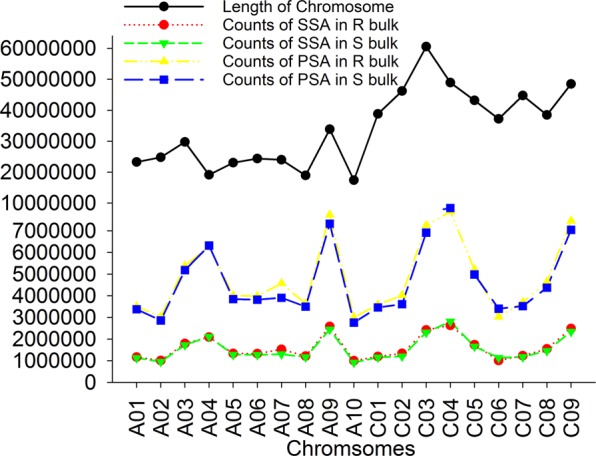
Counts of short reads aligned to chromosomes of the *B*. *napus* reference *B*. *napus* Damor-bzh genome^35^ in single sample alignment (SSA) and pooled sample alignment (PSA), respectively.

The average short read counts and accumulated short read lengths were 30.6 million (M) and 3003 Mb for R bulks, and 29.6 M and 2897 Mb for S bulks, using the SSA method (Table S3). This provided an average depth of coverage for the transcripts of reference genome at 30 folds in R bulks and 29 folds in S bulks. More short reads were aligned to the longer chromosomes A09, C03, C04, and C09, while fewer short reads were assembled in the shorter chromosomes A08 and A10 (Fig. 3). Short reads from three R bulks and three S bulks were further aligned using PSA. As observed previously with the SSA method, more sequences were aligned to A09, C03, C04, and C09, and fewer to A08 and A10 (Fig. 3). A total of 91.9 M short reads were aligned to the *B*. *napus* reference genome, resulting in an alignment of 9008 Mb in length and a coverage of 90 folds of transcripts of the reference genome from the pool of three R bulks, and 88.6 M short reads were aligned, resulting in an alignment 8691 Mb in length and an 87-fold coverage from the pool of three S bulks (Table S3).

### Identification of variants in R and S bulks

**Figure 4:**
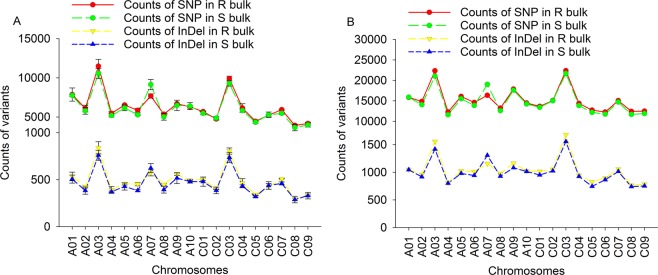
Counts of SNPs and InDels in single sample alignment (SSA) and pooled sample alignment (PSA). (**A**) Counts of SNPs and InDels in SSA method, (**B**) counts of SNPs and InDels in PSA.

About 106K SNPs and 9K InDels were identified in R bulks and 116K SNPs and 9K InDels in S bulks were identified using the SSA method (Table S4). The counts of SNPs and InDels identified using PSA were higher than those using the SSA method, with 288K SNPs and 20K InDels in R bulks and 281K SNPs and 19K InDels in the S bulks (Table S4). There was a strong positive correlation between the numbers of SNPs and InDels (r = 0.97) within R and S bulks using either SSA or PSA. The number of SNPs and InDels varied among chromosomes (Fig. 4), with chromosomes A03 and C03 carrying more variants than others. However, SNPs and InDels seemed to show a similar distribution pattern on different chromosomes among the R and S bulks based on both SSA (Fig. S1) and PSA (Fig. S2).

### Comparison and annotation of variants in R and S bulks

**Figure 5:**
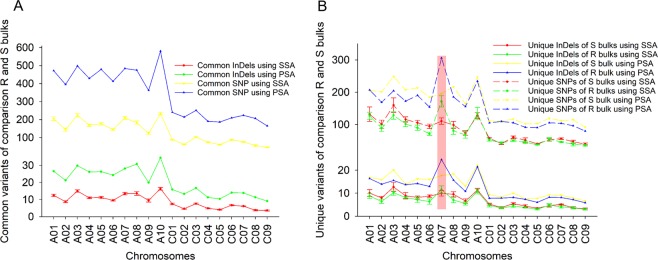
Number of variants (SNPs and Indels) per Mb identified in R and S bulks in comparison with the reference genome of *B*. *napus*. (**A**) Number of monomorphic variants in R and S bulks by SSA and PSA methods. (**B**) Number of plolymorphic variants in R or S bulks by SSA and PSA methods.

Common or monomorphic (mono) variants which were present in both R and S bulks, and unique or polymorphic (poly) variants observed only in R or S bulks were identified. The percentage of mono and poly variants identified using PSA was more than those of SSA on each chromosome, although the ratio of mono and poly variants identified with the two methods were similar (Fig. 5). The mono variants comprised of 51.7% (48.1–54.9%) of the variants identified across the *B*. *napus* genome using PSA, while poly variants comprised of 48.3% (45.1–51.9%) (Table S5). There were no differences among chromosomes in terms of the number of mono and poly variants found in R and S bulks except on chromosome A07, where more poly variants were found in R bulks than in S bulks (Fig. 5B)

**Figure 6:**
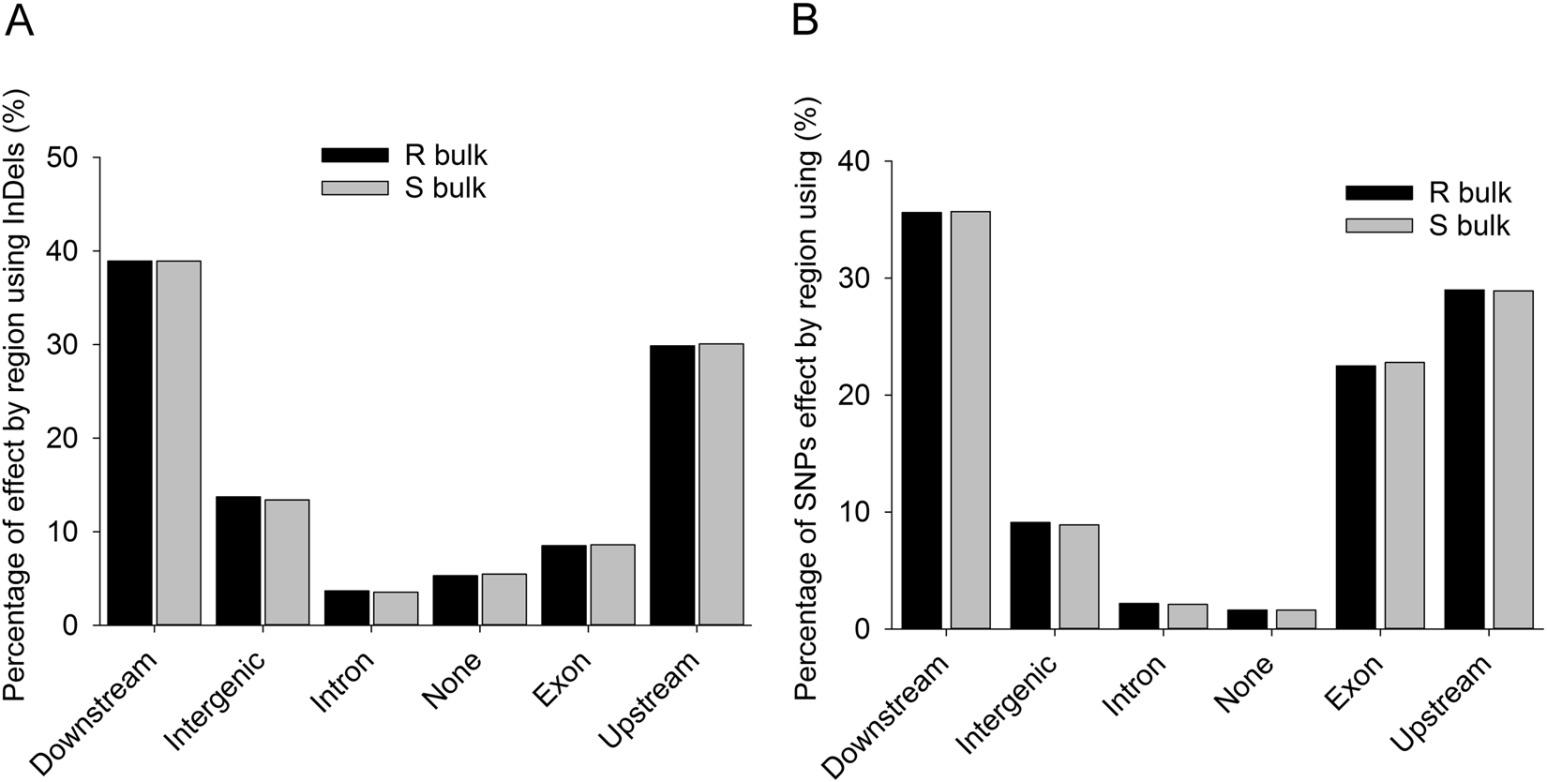
Percentage of variant effects by region using SNPeff analysis. (**A**) Percentage of InDel. (**B**) Percentage of SNP.

In order to investigate the effects caused by the variants, all low-quality sequence reads were filtered out using the *B*. *napus* reference genome^35^. Only the variants identified with PSA were annotated using SNPeff because they included all variants identified through SSA. In total, 18,857 InDels and 272,837 SNPs were annotated in R bulks and 18,816 InDels and 266,772 SNPs in S bulks. The percentage of variant effect by gene regions was almost the same between R and S bulks (Fig. 6); more than 60% of variants were identified in regions down- and up-stream of the gene *Rlm1*, with about 10% of InDels and 20% of SNPs in the gene exon region. Additionally, around 10% of the variants were located in the intergenic region and several variants were in the gene intron region.

### Refining the interval of *Rlm1* using SNP markers identified through BSR-Seq

**Figure 7:**
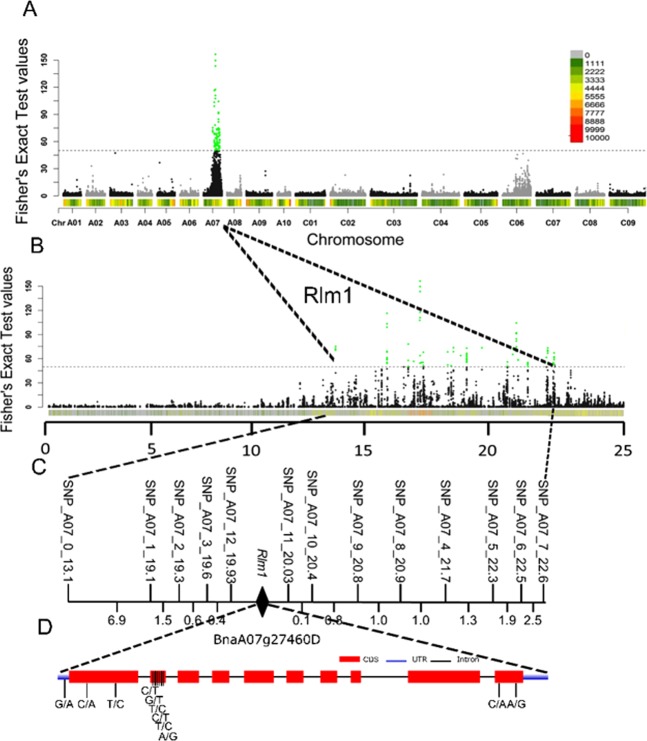
*Rlm1* was located on chromosome A07 using Popoolation2 with window slide (window size = 1000 bp, slide size 100 bp). (**A**) Significant variants by substantial Fisher’s exact values in whole genome (-log (P value) > 8)). (**B**) Significant variants by substantial Fisher’s exact values in chromosome A07 (-log (P value) > 8). Green dots indicate the significant variants in the *Rlm1* interval. (**C**) Fine mapping of *Rlm1* between 19.93 Mb and 20.03 Mb on chromosome A07 using KASP assays with the BC_1_ population (a total of 1,022 plants) and F_2_ population (a total of 2,912 plants). (**D**) *BnaA07g27460D* gene structure and variants.

*Rlm1* was located on chromosome A07 within a region of 13.07 Mb to 22.11 Mb based on significant SNPs (*P* value < 10^−8^; Fst value > 0.2) using modified popoolation2 (Figs 7A,B, Fig. S5). A total of 18,035 variants were identified within this region using PSA, including 16,967 SNPs, 563 insertions and 599 deletions.

In order to narrow down the interval of *Rlm1*, a total of 1,022 BC_1_ plants and 2,912 F_2_ plants, including the 90 R and 90 S plants used for RNA-Seq, were inoculated with the isolate SC006 and genotyped with two flanking SNP markers designed based on SNP information in the *Rlm1* region (Fig. 7C). A total of 13 recombinant lines were identified, and these recombinants were further confirmed to be within the genetic interval with an additional 13 SNP markers (Fig. 7C and Table S1). As a result, *Rlm1* was narrowed down to a 100 Kb region (19.93–20.03 Mb) flanked by markers SNP_A07_12_19.93 and SNP_A07_11_20.03 on chromosome A07 (Fig. 7C). A total of 152 variants (Table S7) could be annotated to 19 *B*. *napus* genes (Table S6) in the *Rlm1* region, including 62 variants identified in both R and S bulks. The variants in the *Rlm1* region were examined using the Integrative Genomics Viewer (IGV)^48^. One gene (*BnaA07g27460D*) showed similar frequencies of DNA variants at about 50% in R bulks and 100% in S bulks (Fig. S5 and Table S7). A total of 11 polymorphic SNPs in *BnaA07g27460D* were identified, including 1 downstream, 9 synonymous, and two missense SNPs. *BnaA07g27460D* (19,986,679–19,989,481 bp) encodes Serine/threonine dual specificity protein kinase with a catalytic domain. The full-length protein sequence was used to search for homologous proteins in the NCBI database, which was aligned using MAFFT and used to construct a neighbor-joining tree with UPGMA clustering (Fig. S6). The results indicated that *BnaA07g27460D* was homologous to *STN7* in predicted genes of *B*. *rapa* and *B*. *oleracea*, and *A*. *thaliana* (Fig. S6).

### Analysis of differential expression genes (DEGs) in R and S bulks

The expression of genes in the *Rlm1* region may provide additional molecular evidence for the confirmation of the candidate *R* gene. A total of 153 DEGs were identified in comparing R bulks with S bulks using cufflink with FDR < 0.05 (Table S8), and18 of them were located within the *Rlm1* region. However, the gene expression level for *BnaA07g27460D* was not significantly different between R and S bulks.

## Discussion

In this study, a BSR-Seq strategy was used to map *Rlm1* to *B*. *napus* chromosome A07 in a 9-Mb region. More than 18,000 variants were identified in this region. Selected SNP loci in the interval were analyzed using KASP assay. A total of 1,022 plants in BC_1_ and and 2,912 plants in F_2_) were analyzed with 13 KASP markers. The *Rlm1* interval was eventually narrowed within an interval of 100 kb through linkage analysis. Previously, *Rlm1* was predicted to be in an interval of approximately 920 kb between a SSR marker (sN9539) and a CAPS marker (Ind07-02) though Blast search^26^. The results indicate that BSR-Seq in combination with KASP assay is much more powerful technology than using marker systems for fine mapping of blackleg resistance genes^49–55^, which marker systems, such as RFLPs^56^, AFLPs^57^, SSRs^58^, CAPSs^59^, and SRAPs^60^, are laborious, time consuming, and low efficiency for identification of QTLs or for marker-assisted selection (MAS). BSR-seq has been used for fine mapping of several resistance genes to clubroot in Brassica species^18,61,62^. However, the application and efficacy of the modified popoolation2 comparing with other mapping by sequencing methods still needs to be determined.

There were more DNA variants identified in chromosomes A03 and C03 than the other chromosomes. The number of variants is usually associated with chromosome length^18^. C03 is the longest chromosome with 60.6 Mb in length in the sequencedreference *B*. *napus* Darmor-bzh genome^35^. In addition, these two chromosomes carry higher numbers of genes although A03 is only 29.8 Mb in length (Table S2).Therefore, it is not surprising that more DNA variants were identified based on BSR-Seq.

In this study, *Rlm1* was located within a range of 100 Kb on chromosome A07. A total of eight blackleg R genes (*LEM1*^5^, *LmFr1*^6^, *LmR1*/*CLmR1*^7,8^, *Rlm1*, *Rlm3*, *Rlm4*, *Rlm7* and *Rlm9*^6–9^) have been reported previously on chromosome A07 (linkage group N7),. *LmR1/CLmR1* was found to co-segregate with three molecular markers, which were located around the 15.8 Mb of chromosome A07, by blasting the marker sequences against the *B*. *napus* genome database^8^. The homologous region of *LEM1* and *LmFr1* cannot be identified based on the genomic information available. The genetic position of *Rlm3*, *Rlm4*, *Rlm7*, and *Rlm9* were clearly different from that of *Rlm1*^9^. In our study, *Rlm1* was fine mapped to an interval between 19.93 Mb and 20.03 Mb with flanking markers SNP9_A07_12_19.93 and SNP8_A07_11_20.03 on chromosome A07, indicating that *Rlm1* is unlikely allelic to *LmR1/CLmR1*.

In the mapping interval, no RLP genes described as blackleg resistance genes^35^ were identified. DNA variants were only identified in gene *BnaA07g27460D* (Fig. S5 and Table S7). Hence, it is possible that *BnaA07g27460D* was a candidate for *Rlm1*. *BnaA07g27460D* is homologous to STN7 in predicted genes of *B*. *rapa* and *B*. *oleracea*, and *A*. *thaliana*. Earlier studies on Arabidopsis clearly demonstrated that thylakoid phosphorylation is predominantly mediated by the protein kinases STN7^63,64^ and STN8^65^. However, several recent studies have revealed that light intensity or quality induces the reduction/oxidation (redox) state of the photosynthetic electron chain^66^. *STN7* has been verified to operate in retrograde signaling through controlling redox balance in the electron transfer chain^67^. These confirmations indicate that *STN7* is involved in plant defense reactions by regulating the excitation of energy distribution between PSII and PSI via the phosphorylation of thylakoid membrane to control cell signaling and systemic response via ROS-induced, hormone-mediated signaling networks^67,68^. In this study, although *BnaA07G27460D* was considered as the candidate gene for *Rlm1*, other candidates may exist. Firstly, the coverage of the sequence reads for several genes in the *Rlm1* region was insufficient for definitive inference of *Rlm1* based only on BSR-Seq. Secondly, the genomic sequence of Darmor-*bzh* was not complete in the *Rlm1* region. Four gaps were found between 19.93 Mb and 20.03 Mb, form 19,922,209 to 19923154 bp, from 19,949,953 to19,960,559 bp, from 19,968,763 to 19,974,252 bp, and from 20,017,902 to 20,020,342. Thirdly, the sequence of Darmor-*bzh* does not carry *Rlm1* gene^69^. Hence, gene cloning and transformation are necessary to confirm the candidate gene of *Rlm1*, but that is out of the scope of the current study. More biochemical and molecular confirmations can be used to confirm the involvement of *STN7* in blackleg resistance.

A total of 153 DEGs were identified in comparing R bulks with S bulks, and18 of them were located within the *Rlm1* region. However, the gene expression level for *BnaA07g27460D* was not significantly different between R and S bulks although DNA variants were identified in the gene. The reason for this is to be determined. No significant difference in R gene expression was previously observed during fungal infection between R and S materials^18^.

Breeding for blackleg resistance in canola is one of the most important objectives in many parts of the world. Use of highly specific markers in MAS could be useful to accurate assess the reaction of lines under controlled conditions. KASP offers cost-effective and scalable flexibility in applications. A large number of SNP sites associated with *Rlm1* were identified based on BSR-Seq. In total, 13 robust SNP markers were confirmed to be associated with *Rlm1* using KASP assay. The closely linked markers shown in Table S7 could be used in MAS for *Rlm1*.

## Supplementary information

Supplementary Figures

Supplementary Table
