# Supplementary material for: Fine mapping of Brassica napus blackleg resistance gene Rlm1 through bulked segregant RNA sequencing
Source: Sci Rep. 2019 Oct 10;9:14600. doi: 10.1038/s41598-019-51191-z (PMC6787231; doi:10.1038/s41598-019-51191-z)
Supplement: Supplementary file 1 — Supplementary Figures [file 41598_2019_51191_MOESM1_ESM.pdf]

Fine mapping of *Brassica napus* blackleg resistance gene *Rlm1* through bulked segregant RNA sequencing

Fuyou Fu[1, 2], Xunjia Liu[1], Rui Wang[1, 3], Chun Zhai[1], Gary Peng, Fengqun Yu[1*], W. G. Dilantha Fernando[2*]

[1]Saskatoon Research Centre, Agriculture and Agri-Food Canada, 107 Science Place, Saskatoon, Saskatchewan, S7N 0X2, Canada

[2]Department of Plant Science, University of Manitoba, Winnipeg, MB R3T 2N2, Canada

[3]Chongqing Engineering Research Center for Rapeseed, College of Agronomy and Biotechnology, Southwest University, Chongqing 400716, China

[*]Corresponding author e-mails: Dilantha Fernando Dilantha.Fernando@umanitoba.ca, Fengqun Yu fengqun.yu@agr.gc.ca

Supplemeantal Figures

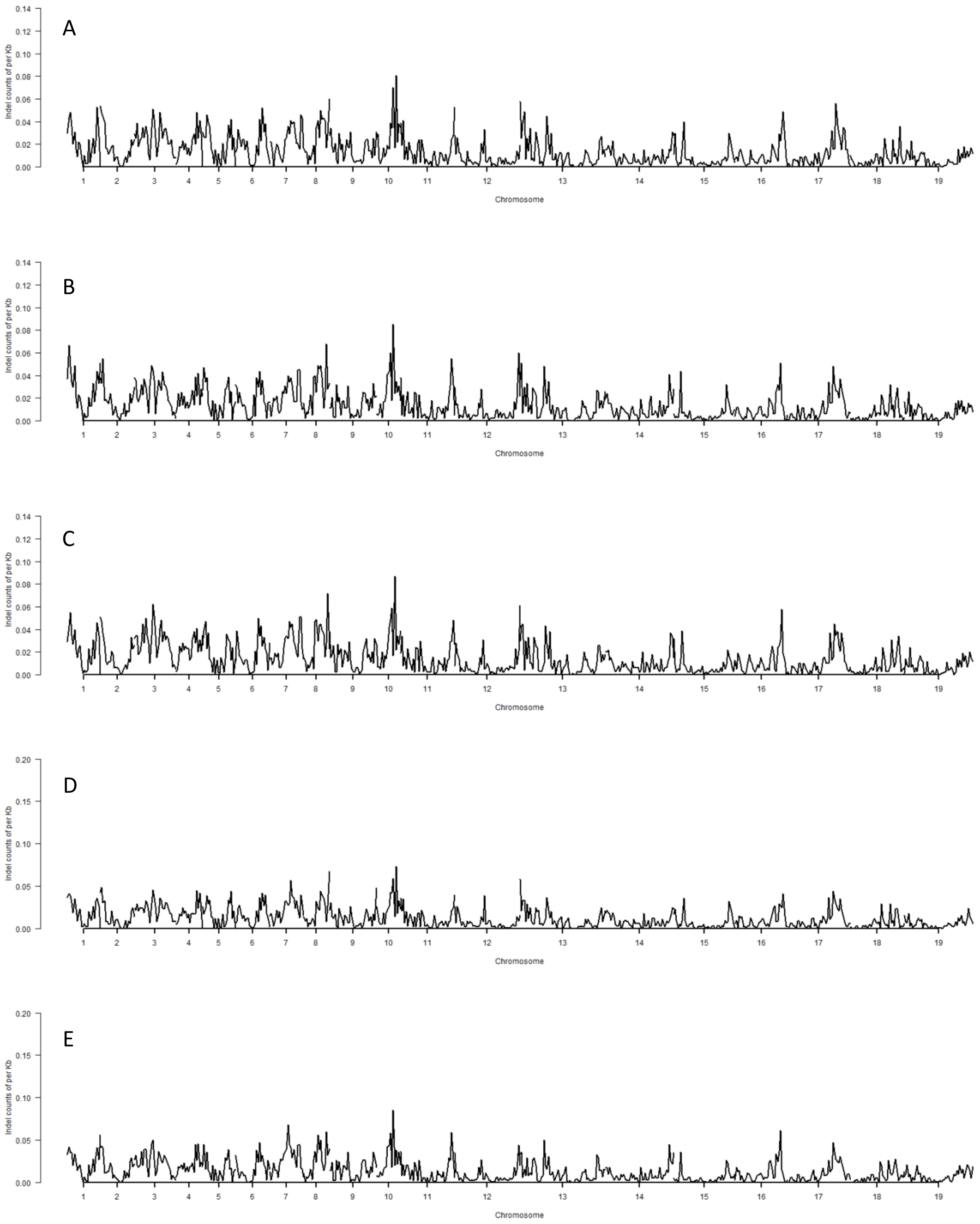
A
B
C
D
E
Indel counts of per Kb
Chromosome
0.00 0.02 0.04 0.06 0.08 0.10 0.12 0.14
0.00 0.05 0.10 0.15 0.20
1 2 3 4 5 6 7 8 9 10 11 12 13 14 15 16 17 18 19

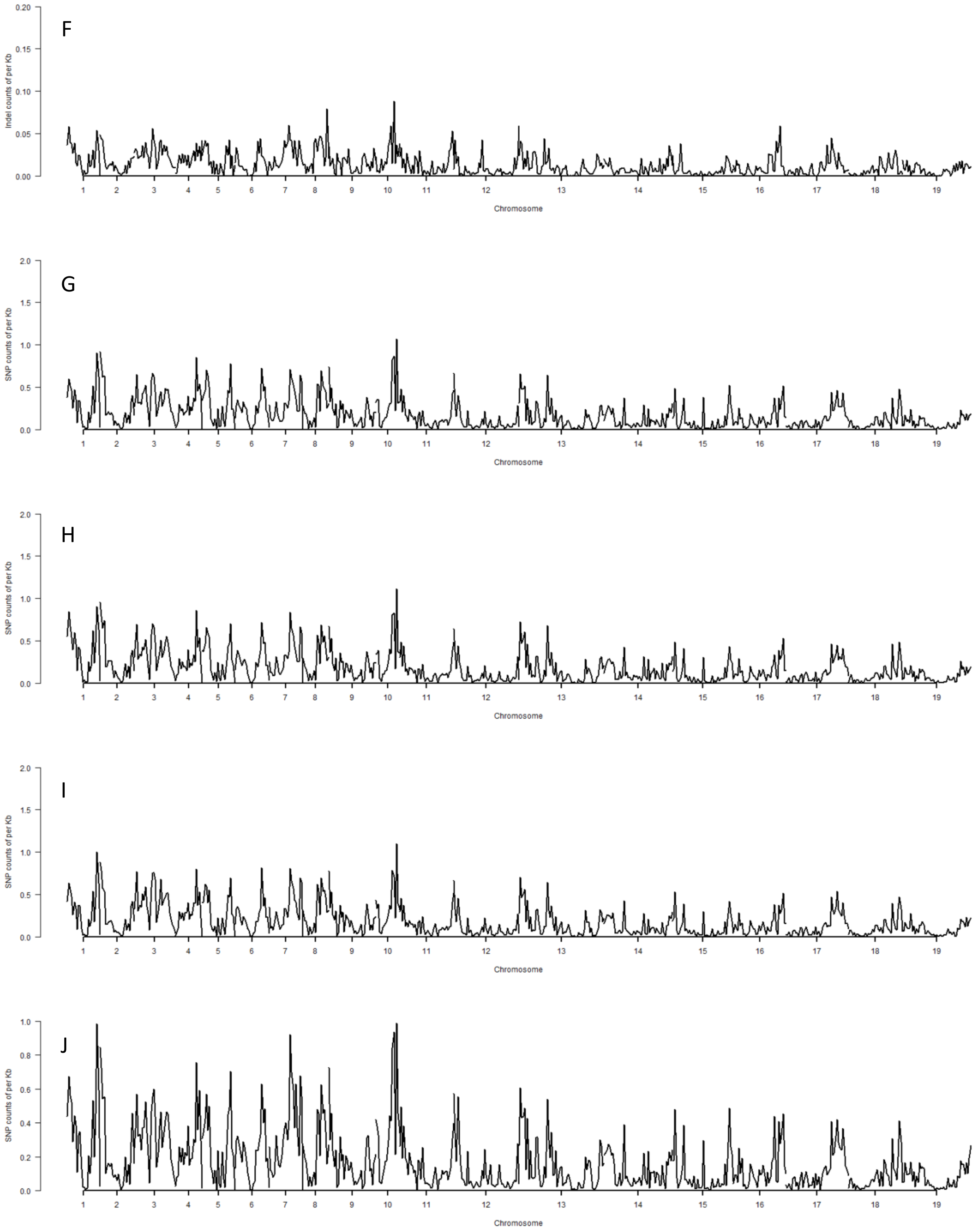
F
0.20
0.15
0.10
0.05
0.00
Indel counts of per Kb
1 2 3 4 5 6 7 8 9 10 11 12 13 14 15 16 17 18 19
Chromosome
G
2.0
1.5
1.0
0.5
0.0
SNP counts of per Kb
1 2 3 4 5 6 7 8 9 10 11 12 13 14 15 16 17 18 19
Chromosome
H
2.0
1.5
1.0
0.5
0.0
SNP counts of per Kb
1 2 3 4 5 6 7 8 9 10 11 12 13 14 15 16 17 18 19
Chromosome
I
2.0
1.5
1.0
0.5
0.0
SNP counts of per Kb
1 2 3 4 5 6 7 8 9 10 11 12 13 14 15 16 17 18 19
Chromosome
J
1.0
0.8
0.6
0.4
0.2
0.0
SNP counts of per Kb
1 2 3 4 5 6 7 8 9 10 11 12 13 14 15 16 17 18 19
Chromosome

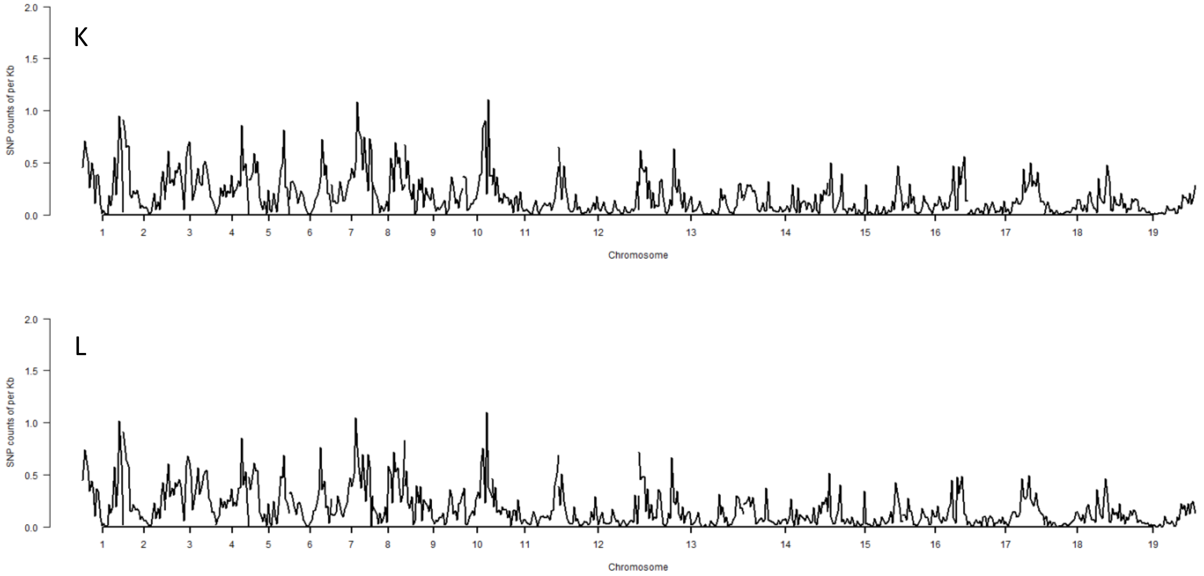

Figure S1. Density of SNPs and InDels in single sample alignment (SSA). A, B, and C show InDels density three replications of R bulks using SSA method. D, E, and F show InDels density three replications of S bulks using SSA method. G, H, and I show SNPs density three replications of R bulks using SSA method. J ,K, and L show SNPs density three replications of S bulks using SSA method. Numeric number 1-19 present the chromosomes A01-A10 and C01-C09 of B. napus genome, repectively.

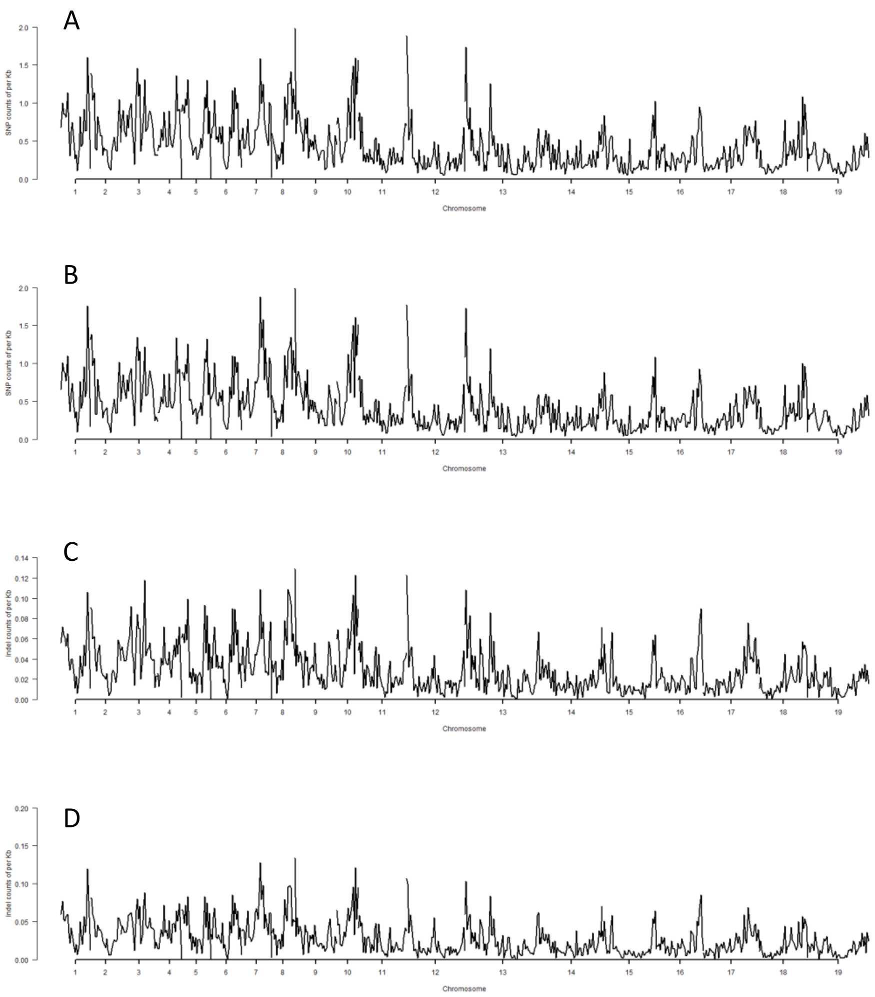

Figure S2. Density of SNPs and InDels in pooled sample alignment (PSA). A and B show SNPs density in R and S bulks using PSA method. C and D show InDels density in R and S bulks using PSA method. Numeric number 1-19 present the chromosomes A01-A10 and C01-C09 of B. napus genome, repectively.

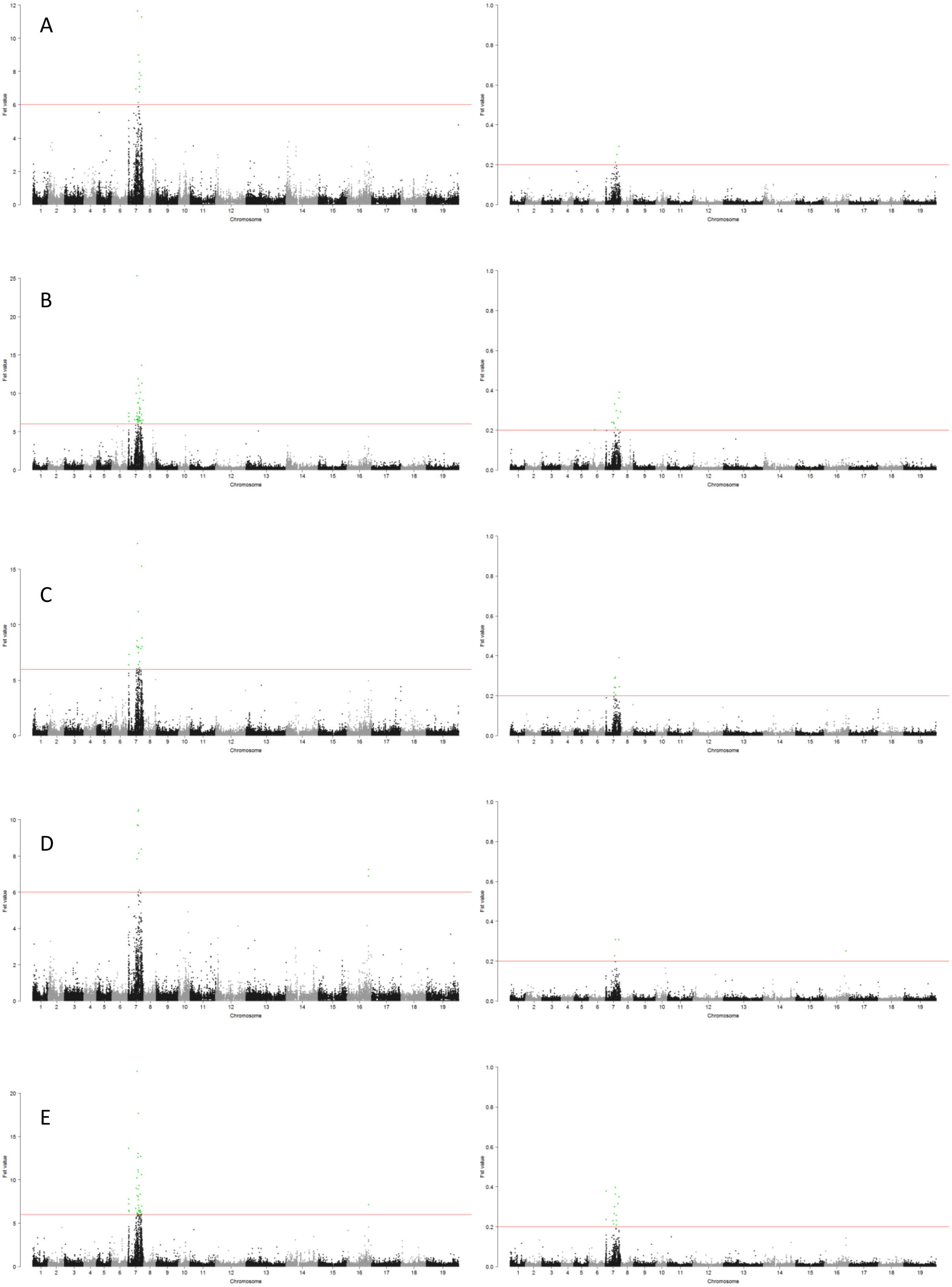
A
B
C
D
E
Fst value
Chromosome

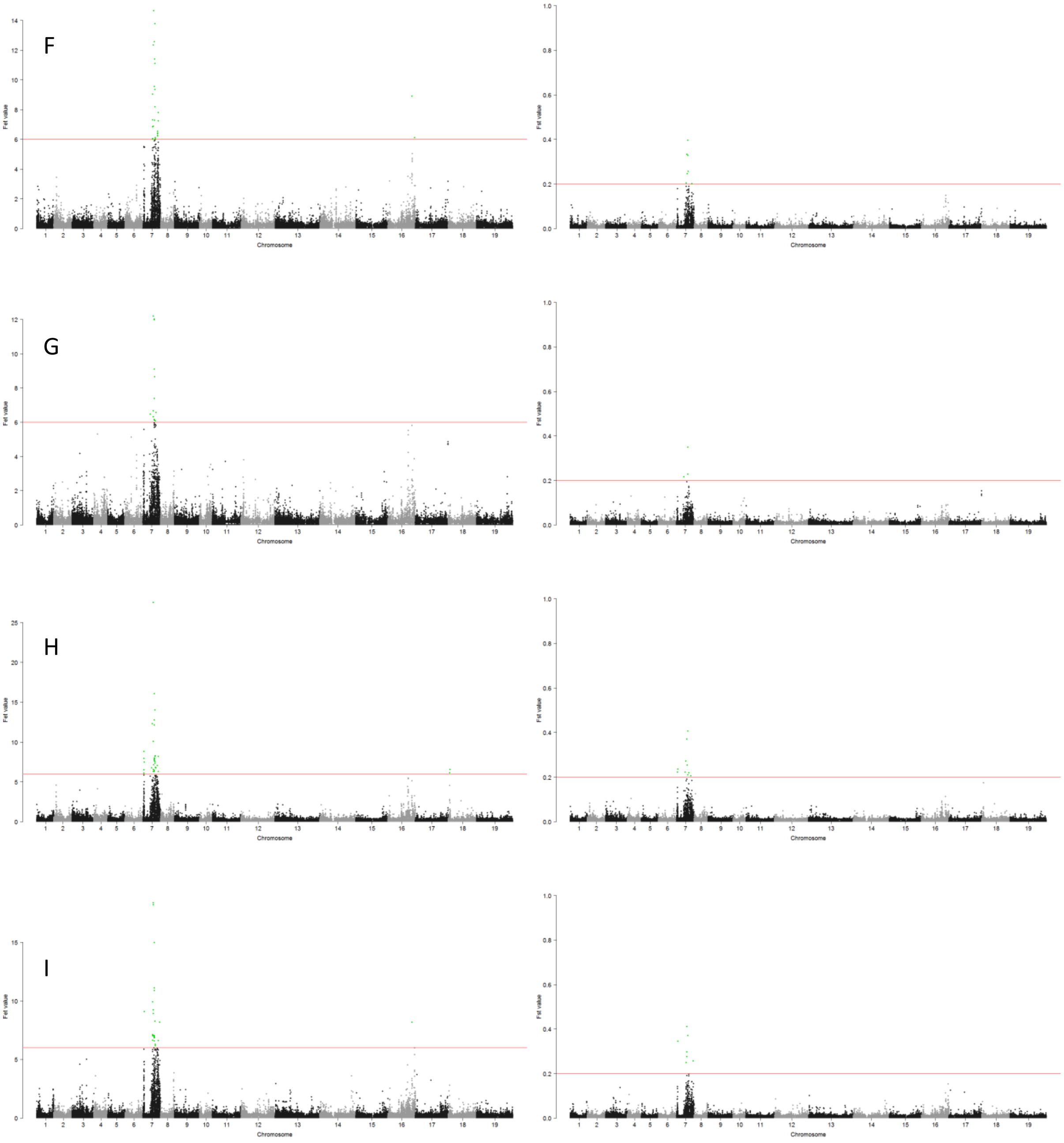

Figure S3.QTL analysis by population 2 with window slide (window size=1000bp, slide size 100bp)using SSA method in R and S bulks. A represents R1 bulk vs S1 bulk. B represents R1 bulk vs S2 bulk. C represents R1 bulk vs S3 bulk. D represents R2 bulk vs S1 bulk. E represents R2 bulk vs S2 bulk. F represents R2 bulk vs S3 bulk. G represents R3 bulk vs S1 bulk. H represents R3 bulk vs S2 bulk. I represents R3 bulk vs S3 bulk. The y axis of left figures show the Fet values, significant value (-log(P value)) is more than 8. The axis of right figures shows the fst values. A significant value (fst value) is more 0.2. Green dots indicate the significant SNP sites in QTL region. Numeric

number 1-19 present the chromosomes A01-A10 and C01-C09 of B. napus genome, repectively.

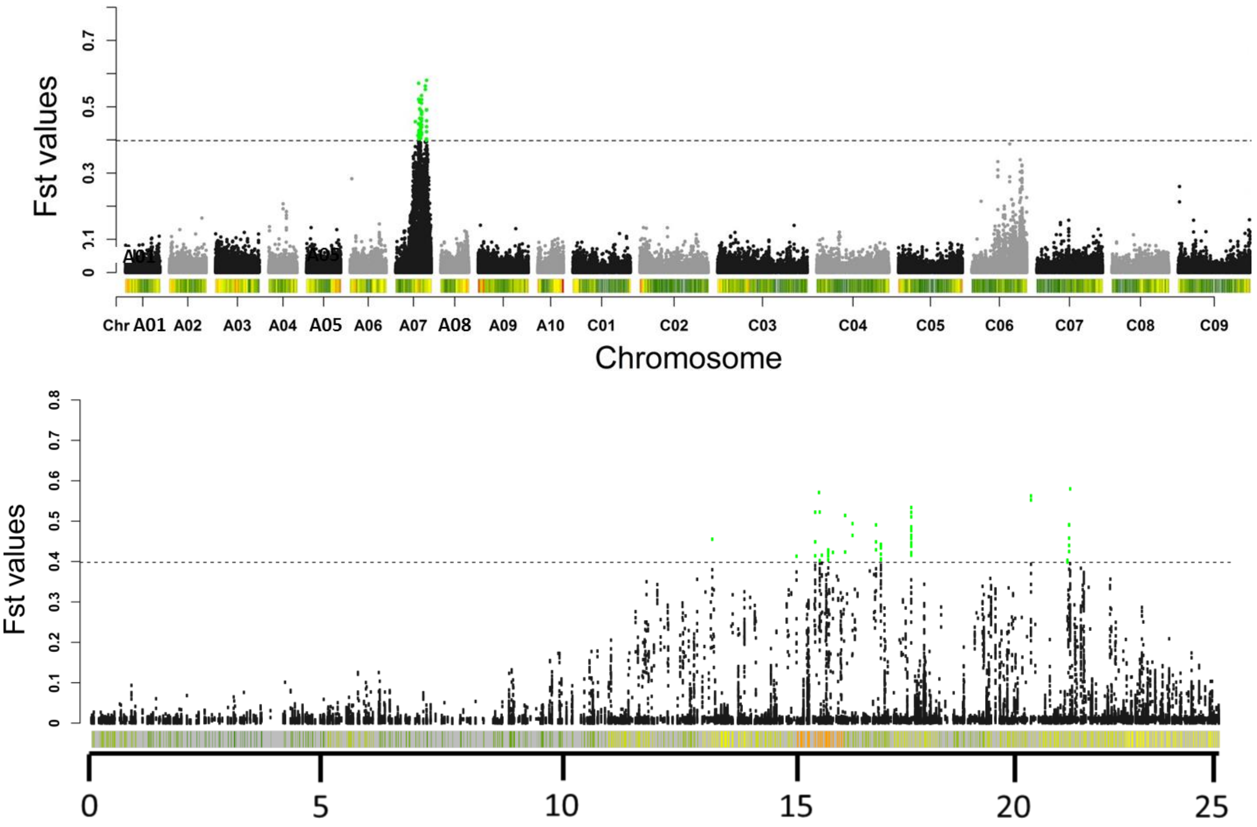

Figure S4. The Rlm1 was located on Chromosome A07 with fst value using Popoolation 2 with window slide (window size=1000bp, slide size 100bp). Green dots present significant variants by fst values with significant value (fst > 0.2).

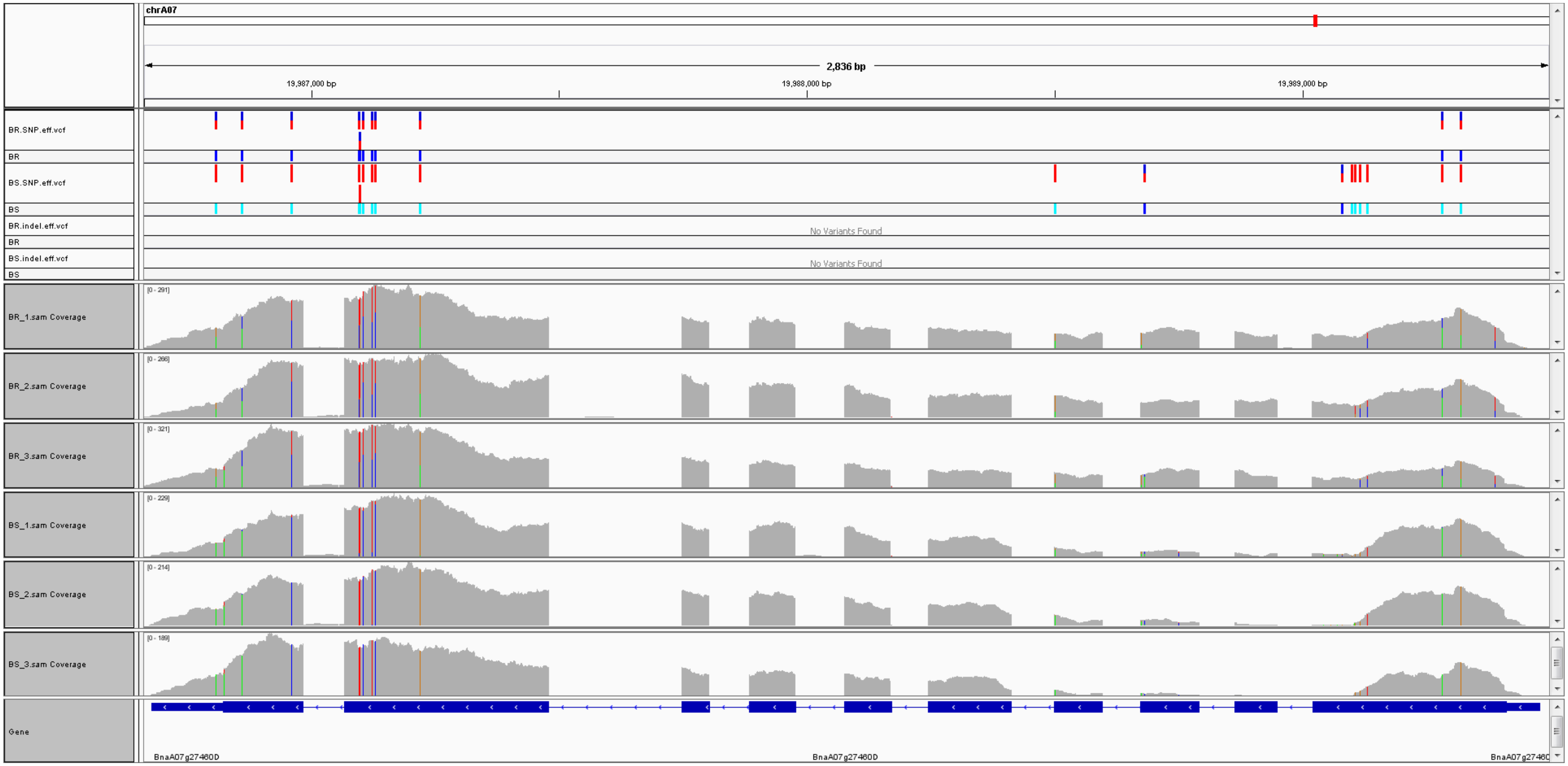

BnaA07G27460D

Figure S5. The sequence variations of candidate genes were examined using Integrative Genomics Viewer (IGV) [1].

1. Thorvaldsdottir H, Robinson JT, Mesirov JP: **Integrative Genomics Viewer (IGV): high-performance genomics data visualization and exploration**. *Brief Bioinform* 2013, **14**(2):178-192.

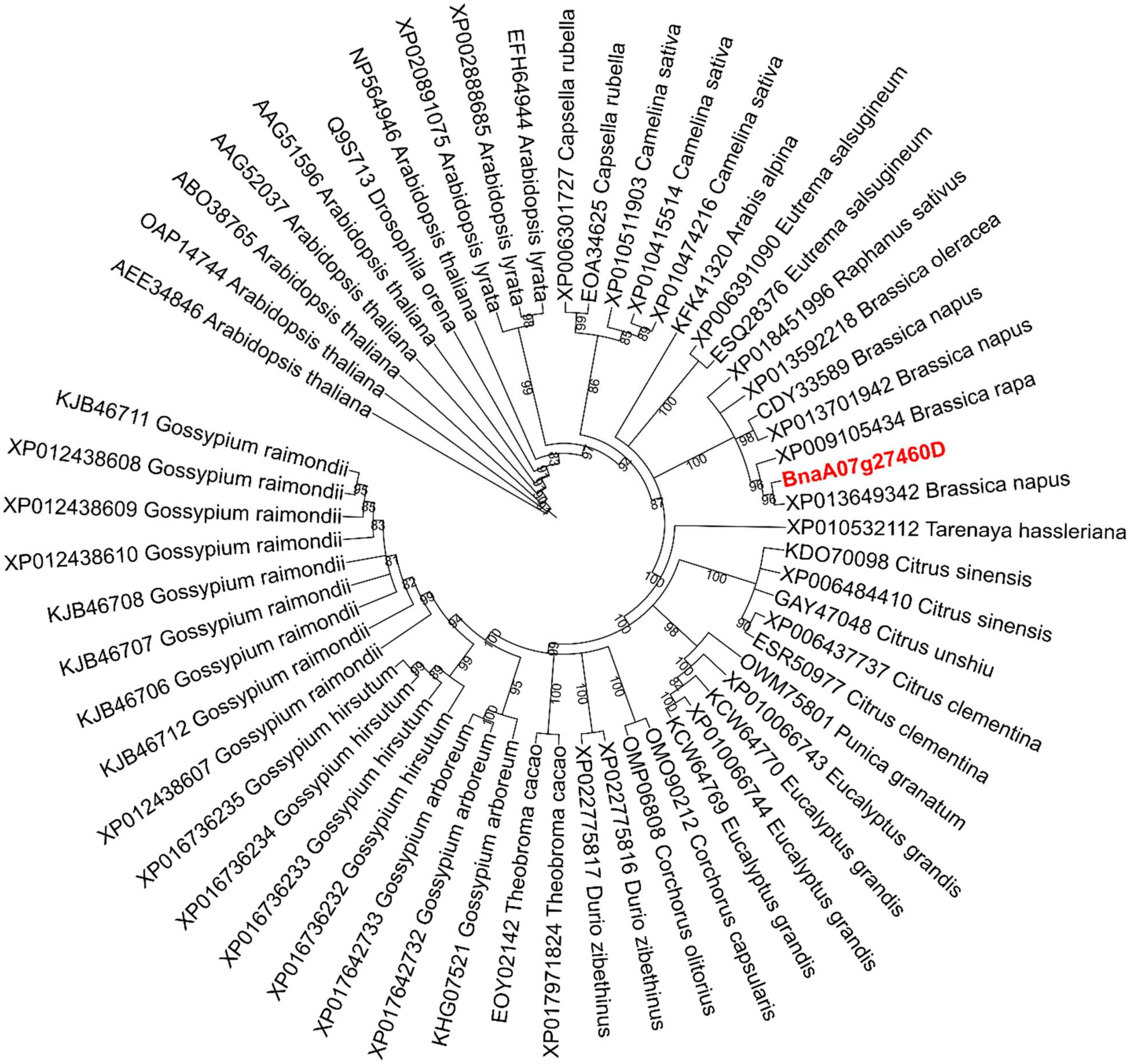

Figure S6. The NJ phylogeny tree of the protein sequence of *BnaA07g27460D* was built using MAFFT with bootstrap 1000. The protein sequences were searched using Blastp with default parameters in the nr database of NCBI.
